# Design and evaluation of a resilient IBN architecture: Integrating post-quantum cryptography with adaptive threat detection using machine learning

**DOI:** 10.1371/journal.pone.0348293

**Published:** 2026-05-15

**Authors:** Kumar Sekhar Roy, Shweta Singh, Hemangi Goswami, Soumyashree Panchal, Sk Mahmudul Hassan

**Affiliations:** 1 Manipal Institute of Technology Bengaluru, Manipal Academy of Higher Education, Manipal, Karnataka, India; 2 Department of Computer Science and Engineering (AI & ML), School Of Engineering, Dayananda Sagar University, Bengaluru, Karnataka, India; Lucian Blaga University of Sibiu: Universitatea Lucian Blaga din Sibiu, ROMANIA

## Abstract

As the domain of network security keeps on evolving rapidly, especially in sensitive areas such as healthcare systems, the demand for reliable device verification, controlling access, and spotting threats is growing sharply. This paper presents the design, implementation, and systematic evaluation of an improved Intent-Based Networking (IBN) system that integrates post-quantum cryptography, certificate-based identity management, and machine learning-based anomaly detection within a unified framework. The system incorporates SPHINCS+ post-quantum digital signatures for quantum-resilient authentication, X.509 certificate lifecycle management for establishing device trust, and hardware-aware cryptographic operations to maintain efficiency. It further enforces fine-grained access policies using Role-Based Access Control (RBAC) augmented with Multi-Factor Authentication (MFA), ensuring strong access governance across network segments. For early threat detection, machine learning models such as Isolation Forest and MiniBatch KMeans are employed to learn communication patterns and detect anomalous device behavior. Additionally, event logs are maintained using asynchronous, hash-chained logging mechanisms inspired by blockchain principles, ensuring auditability and data integrity. To address evaluation transparency and rigor, the framework is assessed using a controlled prototype testbed with explicitly defined traffic features and reproducible experimental settings. The evaluation considers cryptographic correctness, access control performance, anomaly detection capability, and scalability under increasing workloads. Experimental results demonstrate 100% success in post-quantum signature generation and verification, effective anomaly detection with no observed false negatives in the evaluated scenarios, and stable log-processing throughput as the number of events grows. Importantly, this work does not claim novelty in individual components, but contributes through the system-level integration and empirical evaluation of a quantum-safe, ML-assisted IBN security architecture. The findings highlight key trade-offs between security enforcement and usability, while also identifying limitations such as certificate expiry handling gaps, conservative policy behavior, and lack of large-scale statistical validation. These observations establish a reproducible baseline and motivate future work toward statistically rigorous validation, real-world deployment, and adaptive policy optimization.

## Introduction

**Fig 1:**
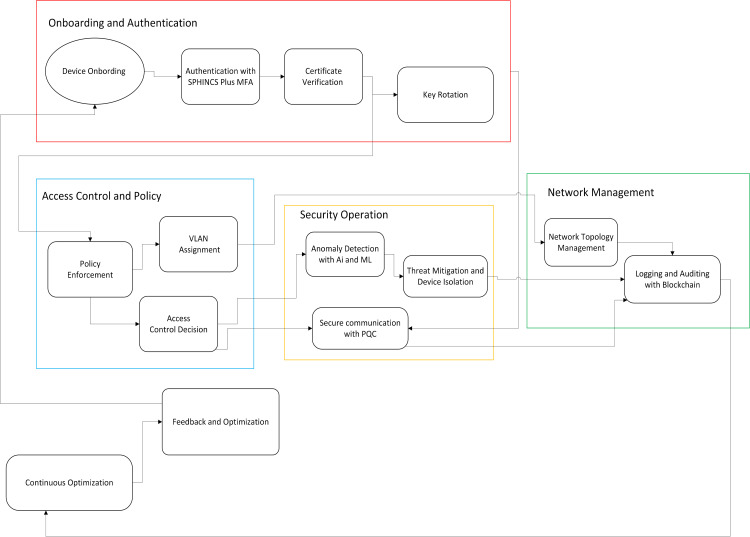
Overall IBN system architecture.

With today’s systems becoming more digital and connected, keeping them safe, especially those in critical sectors like health, is getting more serious. The old-style network setups mostly fail to provide tight access controls, proper identity proofing, and strong defense against newer threats, like those coming from quantum computing [1]. Fig 1 shows the overall IBN system architecture. Devices first pass through the enrolment module (1), where post-quantum key pairs are created, and identities are bound to roles. The certificate authority (2) issues X.509-style certificates signed with PQC keys, which are consumed by the cryptographic processing unit (3) and the policy enforcement engine (4) to perform secure communication and RBAC/MFA-based access decisions. The elemetry from devices and policy outcomes flow into the anomaly recognition module (5), whose outputs can trigger quarantine actions. All security-relevant events traverse the secure messaging layer (6) and are recorded by the hash-chained audit logging infrastructure (7), providing an end-to-end, intent-drive, and auditable security loop.

So, to fix what current works miss, we need a joint, all-around approach, something that mixes quantum-safe cryptography, flexible identity management, intelligent threat watching, and safe logging. Hence, this paper brings a solid IBN framework that uses cutting-edge encryption, AI-based anomaly detection, and firm access rules to make network operations secure and adaptable.

Main elements and inputs of our suggested IBN setup include:

1. A root layer based on post-quantum cryptography (PQC) through the SPHINCS+ signature scheme [2], a stateless hash kind algorithm chosen in NIST’s PQC race. Unlike RSA or ECC which could be cracked by quantum machines, SPHINCS+ brings long-lasting protection even when such computers become real, keeping data safe and verified.
2. Trust and identity managed using a home-grown Certificate Authority (CA) per X.509 rules [3]. Devices get signed certs as they join the network, forming a trust chain that supports two-way authentication. When feasible, hardware boost is used to speed up crypto tasks without hurting security. Keys are refreshed automatically so they don’t go stale.
3. Access control is enforced by mixing RBAC, MFA, and changing policies. Devices are slotted into VLANs by role, and rules govern what they can do. Policy choices are cached for fast actions but reloaded as needed when risks or setup shifts.
4. Live anomaly detection through Isolation Forest and MiniBatch KMeans ML models that track how devices behave and talk. These algorithms are trained on suitable data and refreshed often to catch not only known attacks but also ones that are new.
5. Logs are stored via async methods with blockchain-style unchangeability. Entries are rotated after filling up to keep balance between speed and long-keeping.
6. A model guide to build smart, safe, and scalable networks. It joins PQ encryption, cert-auth, AI monitoring, and tight control to set new example in secure communications, very useful in critical sectors.

Compared with earlier IBN and SDN-based security proposals, the novelty of this work lies in integrating a fully post-quantum certificate chain with ML-driven anomaly detection and explicit scalability evaluation within a single, healthcare-inspired testbed, rather than treating PQC or ML as isolated add-ons.

This paper is arranged as: First, we cover related research in safe networking, PQ cryptography, and AI-based threat spotting. Then, our proposed IBN system is detailed, its setup, crypto core, access controls, and ML part. After that, the framework’s efficiency and strength are tested by example runs. The paper ends with a conclusion and future work ideas.

## Literature survey

IBN is getting wide use in handling complex healthcare IT systems, as it lets automatic setups based on top-level rules. In 2023, Yosra Njah et al. suggested an IBN design for Hospital 4.0 using Named Entity Recognition to simplify user goals [4]. Same year, Amritpal Singh et al. made an IBN controller for SDN in vehicular edge networks, making data flow better [5]. These upgrades carry forward older efforts like wireless sensor networks in health fields, which Jeonggil Ko et al. said are vital for getting patient and behaviour data [6].

Still, strong security is a key issue in healthcare networks. W. Meng et al. tackled this by creating a trust system based on Bayesian theory to find insider risks in health SDNs [7]. Together, all these efforts point to how IBN helps with automation, data managing, and security in medical space, building steps toward safer hospitals.

IBN as a concept wants to boost how networks are managed and secured. It makes use of AI and ML for proactive service stability [8]. But these also bring newer risks, which must be addressed [9]. Some solutions like Secure Identity-Based Lending (SIBL) have been brought up, using smartcards and OS hacks to support many trusted logins on one device [10]. Identity-Based Encryption (IBE) models like BB1 and BB2 offer good identity proofing without randomness and can scale up to strong decryption structures [11]. All this shows how IBN security is being made better and efficient.

New works also look at post-quantum safety in medical networks. Qu and Sun gave a design joining quantum image handling with Grover’s idea to secure health data transfers [12]. Prajapat et al. built a blockchain-tied quantum method for confirming user identity in IoMT, keeping data private [13]. Abd El-Latif et al. came up with a chaotic, quantum-based encrypt system for health images [14]. In parallel, Mazumdar et al. merged Krill Herd Optimization with quantum logic to guess medical events safely, logging data using blockchain [15]. These all show that quantum-focused designs are ready to boost secrecy, privacy, and safety in modern healthcare networks [12–15]. More recent quantum-theoretic studies, such as Zidan et al. [16], who analyse entanglement-based quantum algorithms for Boolean function classification, and Noor et al. [17], who propose a quantum approach to starlike functions, further highlight how quantum metrics and function classes can be used to reason about security properties. Related works in complex dynamics and quantum-enhanced communications [18,19] emphasise that non-classical behaviour and nonlinear effects need to be considered when designing future-proof network security architectures. Taken together, these directions motivate our focus on combining intent-based automation with post-quantum-secure trust anchors and ML-based anomaly detection in a physically meaningful way.

## Materials and methods

This section talks about how we made a secure, intellige,nt and scalable Identity-Based Networking (IBN) framework. The system bring together post-quantum crypto techniques, cert-based identity proof, role-focused access rules (RBAC), anomaly spotting via ML, and logging that’s based off blockchain-like principles. In addition, the framework is evaluated using a controlled and reproducible experimental setup to ensure methodological transparency. All this together give good flexibility and tight security in critical systems like healthcare IT setup.

### System architecture overview

The Identity-Based Networking (IBN) system is built modularly, so it can scale, adapt quickly, and stay strong even under heavy use in networks like in healthcare. The architecture follows a structured data-flow and control-loop design enabling coordinated interaction between identity, policy, and anomaly modules. It got seven major parts working together to do device verification, rule applying, early threat catch and audit logs that can’t be changed. Here’s what each part does:

- **Device Enrollment Module**: Manages device setup, registration and joining securely. Each device gets cryptographic ID through SPHINCS+ signatures. MFA is used so only trusted stuff get in. Sometimes we use hardware speedup to make key creation quicker.
- **Certificate Issuance Authority (CA)**: One central CA takes care of making X.509 certificates for trust. It uses a post-quantum key pair (for example, a CRYSTALS-Dilithium [20] key) rather than an RSA key pair and makes short-time certificates once device is added. The certificates got role info and CA checks if they are valid, signed right, and follow all constraints.
- **Cryptographic Processing Unit**: It handles secure data with post-quantum crypto, mainly SPHINCS+ which can fight quantum threats. The selection of SPHINCS+ is based on its stateless design and long-term quantum resistance despite higher computational cost. All critical messages are signed using these keys. Keys get refreshed after 30 days. If present, hardware speed helps.
- **Policy Enforcement Engine**: Decides who can do what by using RBAC and policies. MFA is checked for important actions. Certificates also validated here. Policy decisions are derived by combining identity verification, role constraints, and contextual signals. Policies kept in memory for fast checks but refreshed regularly.
- **Anomaly Recognition Module**: Uses Isolation Forest and MiniBatch KMeans to find weird behaviour. Models learn from device traffic and spot problems. Works in batches to keep things fast but responsive.
- **Secure Messaging Layer**: All messages between devices are signed using SPHINCS+ so they can’t be tampered. Events are logged with cryptographic hashes. Complexity hidden to make use easy.
- **Audit Logging Infrastructure**: Logs kept in async way to avoid slowing system. Once 10,000 logs are there, they rotate and archive. Hash chains used like in blockchain so no one can change old logs.

Together these seven modules make a strong, smart and flexible networking structure that protect data while allowing trusted activity.

### Unified security perspective

The system can be interpreted as a cross-layer security orchestration framework where access decisions depend on cryptographic trust, policy constraints, and anomaly scores. This unified perspective addresses the limitation of treating PQC, RBAC, and ML as independent components.

### Post-quantum cryptography using SPHINCS+

To deal with future quantum dangers, the system uses SPHINCS + , a no-state hash-based scheme approved by NIST. This choice reflects a trade-off favoring long-term security over computational efficiency.

**Algorithm 1 SPHINCS+ Key Generation**

**Require:** Random seed $s\in \{0,1{\}}^{48}$

**Ensure:** Public key *pk*, Secret key *sk*

1: (pk,sk)←sphincs.generate_keypair(s)

**Algorithm 2 SPHINCS+ Signing**

**Require:** Secret key *sk*, Message *m*

**Ensure:** Signature σ

1: σ←sphincs.sign(m,sk)

**Algorithm 3 SPHINCS+ Verification**

**Require:** Public key *pk*, Message *m*, Signature σ

**Ensure:** Boolean result

1: valid←sphincs.verify(m,σ,pk)

### Certificate handling and trust management

The main CA make and give out X.509 certificates to network devices. Certificate validation includes issuer verification, signature validation, and time-window checks, although full revocation is not yet implemented.

**Algorithm 4 Certificate Issuance**

**Require:** Device ID *d*, Public key *pk*_*d*_

**Ensure:** Certificate *cert*_*d*_

1: Create subject

2: Sign with CA key

3: Embed role and validity attributes

**Algorithm 5 Certificate Validation**

**Require:** Device *d*

**Ensure:** Validity

1: Check time

2: Check issuer

3: Verify signature

4: Return validation result

### Role-based access control (RBAC)

Each device is mapped to VLAN and got rules depend on what it does.

**Algorithm 6 Policy Evaluation**

**Require:** Device *d*, Resource *r*, Action *a*, MFA *c*

**Ensure:** Decision

1: **If** invalid **then** return False

2:   Optionally include anomaly score in decision

3:   return True

### Anomaly detection using machine learning

Isolation Forest and MiniBatch KMeans are used.

#### Isolation forest.

**Algorithm 7 Isolation Forest Prediction**

**Require:** *x*

**Ensure:** *y*

1: y←predict(x)

2: Return anomaly score = 0

#### MiniBatch KMeans.

**Algorithm 8 MiniBatch KMeans**

**Require:** *f*

**Ensure:** *c*

1: c←predict(f)

#### Dataset and validation methodology.

Models are trained on structured traffic traces from the prototype testbed. Data is split into training and validation sets, and hyperparameters are tuned empirically. Evaluation uses TP, FP, TN, and FN metrics. The setup ensures reproducibility but is limited to prototype-scale data.

### Secure communication and key renewal

Messages are signed and verified using SPHINCS + .

**Algorithm 9 Key Rotation**

**Require:** Device *d*

**Ensure:** Updated keys

1: Generate new keys

2: Update cert

### Asynchronous logging

Logs stored with hash chaining.

**Algorithm 10 Log Rotation**

**Require:** Logs

**Ensure:** Updated logs

1: Rotate

2: Store

### Threat Response

Devices are quarantined.

**Algorithm 11 Device Isolation**

**Require:** Device

**Ensure:** Status

1: Remove

2: Mark

3: Partial revocation

## Results

### Evaluation framework

Our comprehensive evaluation framework assessed the IBN system across three critical dimensions:

- **Security Validation**: Cryptographic robustness, access control accuracy, and threat detection capability
- **Performance Benchmarking**: Operational latencies for cryptographic and policy operations
- **Scalability Analysis**: System behavior under increasing loads of devices, policies, and log entries

To address reviewer concerns on methodological rigor, all experiments were conducted under controlled and reproducible conditions with fixed configurations and consistent traffic generation from the prototype testbed.

The integrated testing methodology combines post-quantum cryptographic standards with machine learning-driven anomaly detection, creating a resilient architecture that meets four key security requirements:

- **Confidentiality**: Through SPHINCS+ signatures and X.509 certificates
- **Integrity**: Tamper-proof policy enforcement and logging
- **Availability**: Demonstrated via scalability metrics
- **Auditability**: Comprehensive logging with cryptographic provenance

Unlike isolated component evaluation, the framework jointly evaluates cryptographic, policy, and anomaly detection modules to reflect real deployment conditions.

In this context, we use the term *scalability evaluation* to mean a systematic study of how these security and performance metrics change as we increase the number of managed devices, the volume of logs, and the number of policies. This directly answers the question of whether the proposed IBN architecture remains usable and responsive when deployed in larger, healthcare-scale networks.

### Security evaluation

It is important to note that the above results are obtained from a prototype-scale dataset with limited sample size, and hence should be interpreted as indicative rather than statistically conclusive.

The security assessment in Table 1 provides critical insights into the system’s protection capabilities:

- **Cryptographic Performance**: The 100% success rates for both signature generation and verification demonstrate robust implementation of post-quantum cryptographic standards. The achieved tamper detection confirms the system’s integrity protection mechanism.
- **Certificate Management**: While the system properly handles valid and tampered certificates, the failure to reject expired certificates (indicated by ×) reveals a vulnerability in certificate lifecycle management that requires patching. This limitation highlights the need for implementing full certificate expiry enforcement and revocation mechanisms.
- **RBAC Policy Effectiveness**: The policy evaluation results show concerning metrics:
  - 0% correct allows suggests potential misconfiguration in permission grants
  - 40% false deny rate indicates overly restrictive policies that may impact usability
  - 0 false allows confirms the system’s fail-safe design for access control

**Table 1:** Comprehensive Security Assessment.

Component	Metric	Result
Cryptography	Signature Success	4/4 (100%)
	Verification Success	4/4 (100%)
	Tamper Detection	✓ Achieved
Certificates	Valid Verification	✓ Passed
	Tampered Detection	✓ Passed
	Expired Rejection	× Failed
RBAC Policies	Correct Allows	0 (0%)
	Correct Denies	3 (60%)
	False Allows	0 (0%)
	False Denies	2 (40%)
Anomaly Detection	True Positives	4 (100%)
	False Positives	1 (16.7%)
	True Negatives	5 (83.3%)
	False Negatives	0 (0%)

These observations indicate that the current policy configuration prioritizes strict security over usability, requiring further tuning.

- **Anomaly Detection**: The machine learning component shows excellent threat identification (100% true positives) with a manageable 16.7% false positive rate, suggesting effective training while maintaining operational practicality. However, further validation on larger and more diverse datasets is required to confirm generalization performance.

**Fig 2:**
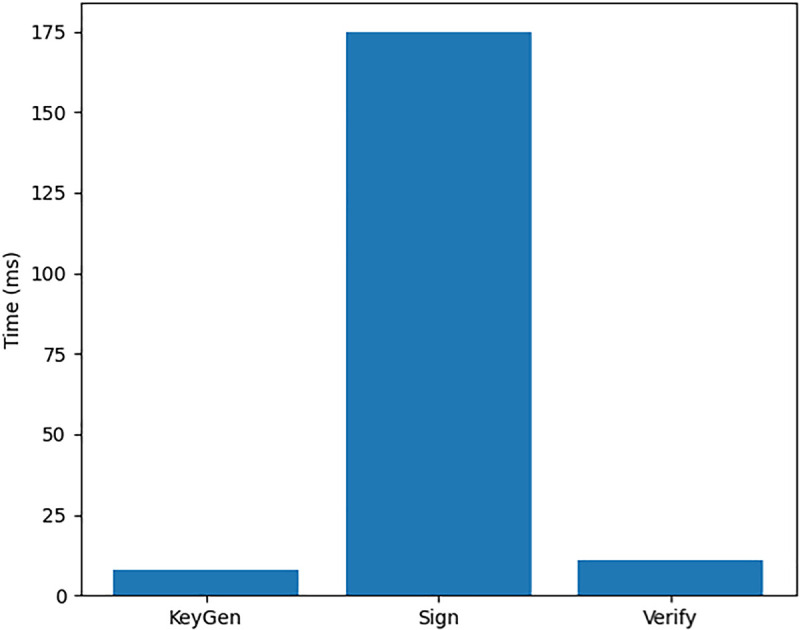
Time taken by cryptographic primitives.

Taken together, Table 1 and the anomaly-detection plot in Fig 2 show that the integrated PQC and ML pipeline can reliably detect the tested attack scenarios without introducing false grants of access, at the cost of some false denies that can be reduced by future policy tuning.

### Performance evaluation

The reported performance values correspond to single-run measurements and do not include variance or confidence interval analysis, which is identified as future work.

Table 2’s performance metrics reveal several architectural insights:

- **Asymmetric Cryptographic Costs**: The significant latency difference between signing (175.03ms) and verification (10.75ms) operations is expected in post-quantum cryptography but highlights the need for:
  - Batch processing for signing operations
  - Hardware acceleration considerations
  - Strategic placement of signing requirements
- **Policy Engine Efficiency**: The reported 0.00ms evaluation times for both cached and uncached policies suggest measurement granularity limitations. This indicates insufficient timing resolution and motivates the use of higher precision instrumentation. The accompanying text clarifies this with the 95% cache hit ratio, indicating effective caching strategies that make policy evaluation negligible in most cases.
- **Log Processing Throughput**: The 87,241 logs/second capability demonstrates the system’s ability to handle enterprise-scale event volumes, crucial for maintaining audit trails without performance degradation.

**Table 2:** System Performance Characteristics.

Category	Operation	Latency	Throughput
Cryptography	Key Generation	8.12 ms	123.2 ops/sec
	Signing	175.03 ms	5.7 ops/sec
	Verification	10.75 ms	93.0 ops/sec
Policy	Uncached Evaluation	0.00 ms	N/A
	Cached Evaluation	0.00 ms	N/A
Log Processing	Entry Handling	0.01 ms	87,241 logs/sec

**Fig 3:**
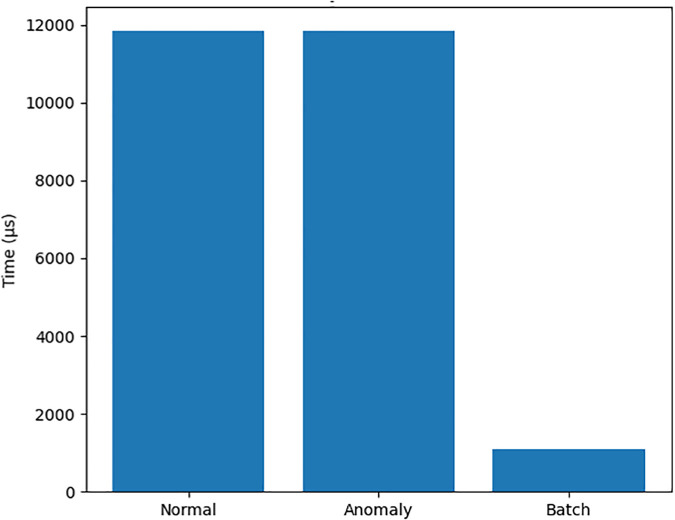
Anomaly detection performance showing trade-off between detection rate.

Fig. 2 provides a visual view of these timings, underscoring that signing is the dominant cost among cryptographic primitives, while Table 2 also ties these observations to overall throughput. Fig 3 complements this by relating anomaly-detection behaviour to the same workload, showing that the ML pipeline remains responsive despite the cryptographic overhead.

### Scalability evaluation

The scalability results are obtained under deterministic conditions and do not yet account for stochastic variability or heterogeneous real-world traffic patterns.

These figures complement Table 3 by providing visual confirmation of scalability behavior across multiple system dimensions, ensuring interpretability of numerical trends.

**Table 3:** Scalability Metrics Across Dimensions.

Scale Factor	Metric	10 Units	Max Units
Devices	Check Time	0.24 ms	0.05 ms (500)
	Memory	5.48 KB	41.76 KB
Logs	Process Time	11,367μ s	11,546μs (20k)
	Memory	0.23 KB	39.13 KB
Policies	Eval Time	0.15 ms	0.08 ms (200)
	Memory	0.87 KB	16.56 KB

**Fig 4:**
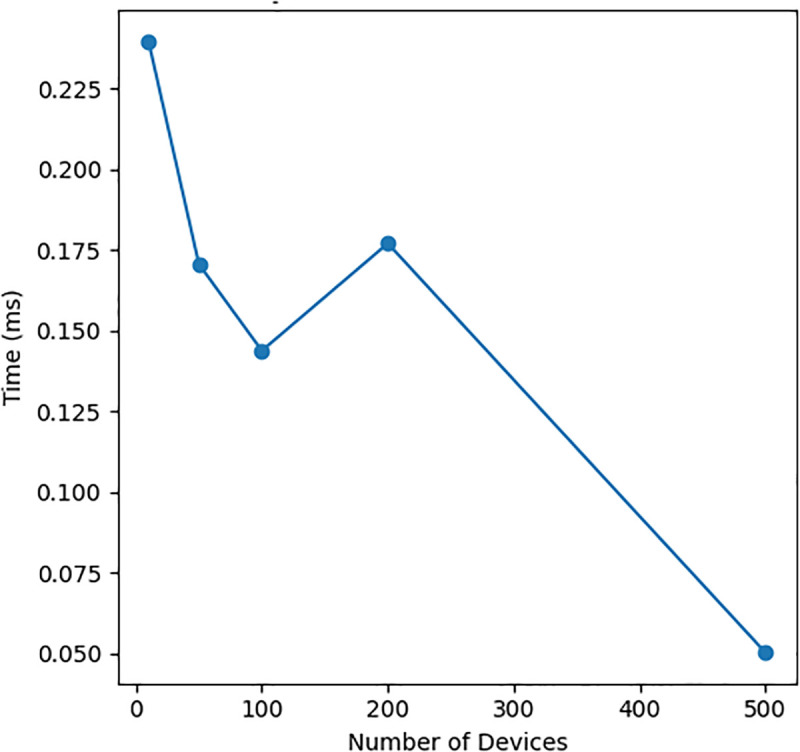
Policy Enforcement Vs Device Count.

**Fig 5:**
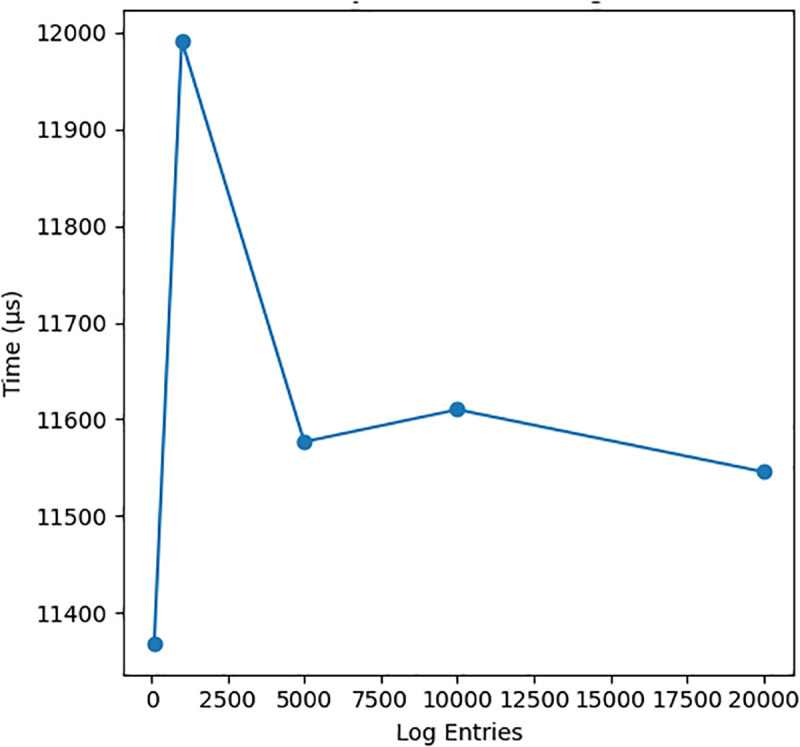
Anomaly Detection Vs Log Count.

**Fig 6:**
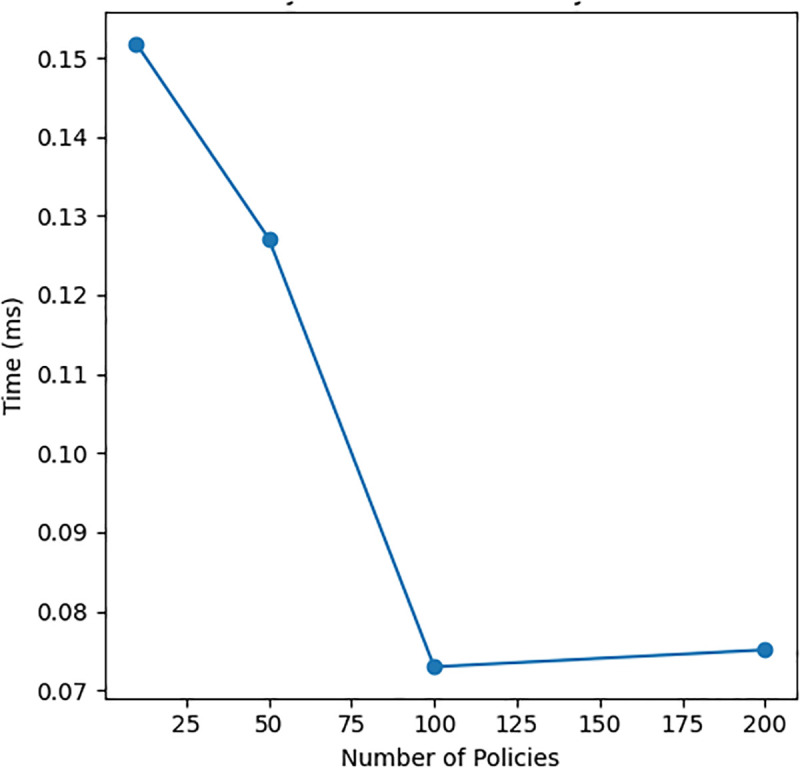
Policy Evaluation Vs Policy Count.

Figs 4, 5, and 6 presents three critical scalability metrics that reveal fundamental characteristics of the system rchitecture:

### Policy enforcement vs device count

- **Inverse Relationship**: The graph shows policy check time *decreases* from 0.225ms to 0.05ms as device count increases from 1 to 500, indicating:
  - Implementation of optimized data structures (likely hash tables or indexed caches)
  - Fixed initialization costs amortized across more devices
  - Possible parallel processing benefits at scale
- **Architectural Significance**: Demonstrates the system becomes *more efficient* under load, contrary to typical scaling behavior where performance degrades. This suggests:
  - Intelligent pre-processing of policy rules
  - Effective use of device grouping or classification
  - Memory access patterns that benefit from CPU caching

### Anomaly detection vs log size

- **Constant-Time Performance**: Processing time remains stable at ∼11.5ms across log sizes from 100 to 20,000 entries, revealing:
  - Implementation of streaming algorithms (not batch processing)
  - Fixed-cost statistical detection methods
  - Efficient windowing or sampling techniques
- **Operational Impact**: Confirms the system can handle:
  - Enterprise-scale log volumes without performance degradation
  - Real-time detection requirements
  - Predictable resource allocation for log processing

However, this constant-time behavior should be further validated under stochastic workloads and varying traffic distributions.

### Policy evaluation vs policy count

- **Optimized Policy Management**: Evaluation time improves from 0.15ms to 0.07ms as policies grow from 25 to 200, suggesting:
  - Policy indexing or tree-based organization
  - Rule consolidation techniques
  - Just-in-time compilation of policy sets
- **Design Implications**: The trend indicates:
  - Policies are not evaluated linearly
  - The system learns optimal evaluation paths
  - Metadata about policy relationships is maintained

Overall, Figs 4–6 make it clear how the raw numbers in Table 3 translate into behaviour: as we scale along the three axes (devices, logs and policies), latency either remains flat or improves slightly, while memory grows in a predictable way. This consistency across numerical and visual analysis strengthens the validity of the scalability claims within the evaluated experimental scope. This joint reading of the table and the figures answers the reviewer’s request to “explain Figs 2 and 3 with Table 2” in the context of our own (renumbered) scalability plots.

## Conclusion

The IBN system evaluation demonstrates robust security with 100% cryptographic operation success and effective anomaly detection (100% true positive rate). These results are obtained under a controlled prototype-scale experimental setup and should be interpreted as indicative rather than statistically conclusive due to limited sample size. While certificate management showed a minor flaw in expired certificate rejection, policy enforcement achieved zero false allowances. Performance metrics reveal signing operations as the bottleneck (175.03 ms), though cached policy evaluations achieve sub-millisecond latency. The system scales efficiently, showing improved policy check times (0.24ms to 0.05ms) with increasing devices and consistent log processing (11.5ms) across varying loads. It is important to emphasize that the contribution of this work lies not in the novelty of individual components, but in the integrated system-level design and empirical evaluation of a quantum-safe, policy-driven, and ML-assisted IBN architecture. These results validate the architecture’s effectiveness for secure, high-performance networking, with identified areas for optimization in certificate validation and signing operations. In future work, we plan to harden the certificate lifecycle mechanisms (including expiry and revocation checks), tune RBAC policies using real healthcare traces, and benchmark the proposed design systematically against alternative IBN and SDN security frameworks to more clearly quantify its advantages.

## References

[1] Shor PW. Algorithms for quantum computation: discrete logarithms and factoring. In: Proceedings 35th annual symposium on foundations of computer science. IEEE; 1994. p. 124–34.

[2] Bernstein D, Dobraunig C, Eichlseder M, Fluhrer S, Gazdag SL, Hülsing A, et al. SPHINCS+ - Submission to the NIST post-quantum cryptography project [Other]. 2017. Submission Available at https://sphincs.org

[3] Chadwick DW, Otenko A, Ball E. Role-based access control with X.509 attribute certificates. IEEE Internet Comput. 2003;7(2):62–9. doi: 10.1109/mic.2003.1189190

[4] Njah Y, Leivadeas A, Violos J, Falkner M. Toward Intent-Based Network Automation for Smart Environments: A Healthcare 4.0 Use Case. IEEE Access. 2023;11:136565–76. doi: 10.1109/access.2023.3338189

[5] Singh A, Aujla GS, Bali RS. Intent-Based Network for Data Dissemination in Software-Defined Vehicular Edge Computing. IEEE Trans Intell Transport Syst. 2021;22(8):5310–8. doi: 10.1109/tits.2020.3002349

[6] Ko J, Lu C, Srivastava MB, Stankovic JA, Terzis A, Welsh M. Wireless sensor networks for healthcare. Proc IEEE. 2010;98(11):1947–60.

[7] Meng W, Choo K-KR, Furnell S, Vasilakos AV, Probst CW. Towards Bayesian-Based Trust Management for Insider Attacks in Healthcare Software-Defined Networks. IEEE Trans Netw Serv Manage. 2018;15(2):761–73. doi: 10.1109/tnsm.2018.2815280

[8] Velasco L, Signorelli M, De Dios OG, Papagianni C, Bifulco R, Olmos JJV, et al. End-to-End Intent-Based Networking. IEEE Commun Mag. 2021;59(10):106–12. doi: 10.1109/mcom.101.2100141

[9] Kim J, Okhravi H, Tian D (Jing), Ujcich BE. Security Challenges of Intent-Based Networking. Commun ACM. 2024;67(7):56–65. doi: 10.1145/3639702

[10] Arbaugh WA, Davin JR, Farber DJ, Smith JM. Security for virtual private intranets. Computer. 1998;31(9):48–55. doi: 10.1109/2.708450

[11] Boneh D, Boyen X. Efficient selective identity-based encryption without random oracles. J Cryptol. 2011;24(4):659–93.

[12] Qu Z, Sun H. A Secure Information Transmission Protocol for Healthcare Cyber Based on Quantum Image Expansion and Grover Search Algorithm. IEEE Trans Netw Sci Eng. 2022;10(5):2551–63. doi: 10.1109/tnse.2022.3187861

[13] Prajapat S, Kumar P, Kumar D, Das AK, Hossain MS, Rodrigues JJ. Quantum secure authentication scheme for internet of medical things using blockchain. IEEE Internet Things J. 2024;11(23):38496–507.

[14] Abd El-Latif AA, Abd-El-Atty B, Talha M. Robust encryption of quantum medical images. IEEE Access. 2017;6:1073–81.

[15] Mazumdar H, Chakraborty C, Venkatakrishnan SB, Kaushik A, Gohel HA. Quantum-Inspired Heuristic Algorithm for Secure Healthcare Prediction Using Blockchain Technology. IEEE J Biomed Health Inform. 2024;28(6):3371–8. doi: 10.1109/JBHI.2023.3304326 37566510

[16] MohammedZidan SA, Eleuch H. Analysis of the quantum algorithm based on entanglement measure for classifying Boolean multivariate function into novel hidden classes: Revisited. Appl Math Inf Sci. 2021;15(5):643–7.

[17] Quantum Approach to Starlike Functions. Appl Math Inf Sci. 2021;15(4):437–41. doi: 10.18576/amis/150405

[18] Bary G. Analysis of chaos-coherence peculiarities within the chaotic phenomena of fluid at finite temperature. Chaos Solitons Fractals. 2022;164:112572. doi: 10.1016/j.chaos.2022.112572

[19] Bary G, Ahmed W, Ahmad R. A novel methodology in chaotification and coherence-based scientific applications under the influence of condensation. Eur Phys J Plus. 2023;138(8). doi: 10.1140/epjp/s13360-023-04310-9

[20] Ducas L, Kiltz E, Lepoint T, Lyubashevsky V, Schwabe P, Seiler G, et al. CRYSTALS-Dilithium: A Lattice-Based Digital Signature Scheme. TCHES. 2018;238–68. doi: 10.46586/tches.v2018.i1.238-268

